# Differential mRNA and miRNA Profiles Reveal the Potential Roles of Genes and miRNAs Involved in LPS Infection in Chicken Macrophages

**DOI:** 10.3390/genes12050760

**Published:** 2021-05-17

**Authors:** Qi Zhang, Jie Wang, Jin Zhang, Jie Wen, Guiping Zhao, Qinghe Li

**Affiliations:** State Key Laboratory of Animal Nutrition, Institute of Animal Sciences, Chinese Academy of Agricultural Sciences, Beijing 100193, China; zhangq0117@126.com (Q.Z.); iwangjie0226@163.com (J.W.); zhangjin0913@126.com (J.Z.); wenjie@caas.cn (J.W.); zhaoguiping@caas.cn (G.Z.)

**Keywords:** chicken, HD11, lipopolysaccharide, gene, miRNA

## Abstract

Lipopolysaccharide (LPS) is a component of the cell wall of Gram-negative bacteria, and triggers an inflammatory response both in vitro and in vivo. Here, we used LPS from *Escherichia coli* serotype enteritidis to stimulate chicken macrophages (HD11) and conducted the transcriptome analysis using a bioinformatics approach to explore the functions of immune-related genes and miRNAs. In total, 1759 differentially expressed genes (DEGs) and 18 differentially expressed (DE)-miRNAs were detected during LPS infection. At 6 h post infection, 1025 DEGs and 10 miRNAs were up-regulated, and 734 DEGs and 8 DE-miRNAs were down-regulated. Based on both RNA hybrid and miRanda systems, 55 DEGs could be targeted by 14 DE-miRNAs. The target genes were related to the immune response, such as *IRF8*, *STAT3*, *TRAF7*, and other potential candidate genes. The DE-miRNAs miR146a-3p, miR6583-5p, and miR30c-2-3p were investigated further. They were predicted to target 34 genes that may also be candidates for immune-related miRNAs and genes. Our results enhanced our understanding of the pathogenic mechanisms of Gram-negative bacteria in chickens.

## 1. Introduction

LPS (lipopolysaccharide), also known as endotoxin, is a highly acylated glycolipid on the cell surface of Gram-negative bacteria. LPS is composed of three parts, a highly acylated di-glucosamine backbone (lipid A) connected to a polysaccharide containing repeating sugars (O-antigen) and linked through a highly conserved oligosaccharide Kdo/heptose core [1,2]. Lipid A can represent its physiological role, and is considered a manifestation of the toxic effects of Gram-negative bacterial infection [3]. Early work on LPS began in the 1960s on *Escherichia coli* and *Salmonella typhimurium*. Subsequently, the structure of LPS was elucidated [4], and its physiological function was revealed. LPS is now recognized as a primary component of the cell wall and the primary cause of the host cell infection induced by Gram-negative bacteria. LPS has been used in models of the pathogenesis of infections with *Salmonella* and *Escherichia coli*.

LPS can stimulate the host cell to trigger an inflammatory response. The main pathway is binding to the transmembrane protein, Toll-like receptor (TLR), located on the surface of many immune cells [5]. In mammalian cells, LPS binds with LPS binding protein (LBP) and facilitates the association between LPS and CD14. CD14 is a receptor for complexes of LPS and LBP. It transfers LPS to the TLR4/MD-2 receptor complex to induce inflammation and produce a series of cytokines and bioactive molecules, causing a series of inflammatory events [6]. Inflammation is a typical defense response to pathogens, mainly mediated by activated macrophages, mononuclear cells, and other immune cells [7,8]. These immune cells produce various inflammatory factors or immune effector molecules, such as tumor slippage factor, interleukin (IL)-6, nuclear factor (NF)-κB, and IL-1β, which participate in the initiation and development of inflammation and promote inflammation amplification, thus achieving the purpose of removing stimulating factors [9,10,11]. 

MicroRNAs (miRNAs) are small non-coding RNAs that regulate gene expression via either translational repression or mRNA degradation at the posttranscriptional level. The miRNAs are involved in various biological processes, such as gene imprinting, cell differentiation, proliferation, apoptosis, and tumorigenesis [12,13,14,15]. The miRNAs carry out their regulatory function mainly through binding to the 3′-untranslated region (UTR) of target genes. The 2–8 bases at the 5′ terminus of mature miRNAs are known as the seed region, and are highly conserved in sequences among different organisms [16]. Emerging evidence demonstrates that miRNAs are critical regulators of inflammatory responses, host–pathogen interactions, and lipoprotein formation and secretion. In murine macrophages, miRNA let-7 targets Tet methylcytosine dioxygenase 2 (Tet2) to promote IL-6 levels, thus characterizing a regulatory pathway in which a miRNA acts as a feedback inhibitor of inflammatory processes [17]. TRAF6 has been identified as a downstream target of miR-140 and is negatively modulated by miR-140, thereby promoting *Mycobacterium tuberculosis* survival and reducing proinflammatory cytokines in macrophages [18]. miR155 and miR1306 affect the expression of downstream cytokines by targeting key genes in the TLR4 pathway. For example, miR1306 can inhibit the production of proinflammatory cytokines such as NF-κB and IL-1β [19,20].

In this study, we analyzed the expression patterns of mRNA and miRNA in the biological responses of HD11 cells to LPS from *E. coli*. The expression differences in genes and miRNAs during the inflammatory response were investigated. The miRNA-gene target pairs that might be related to immunological processes were identified. The results provide new insights into the potential role of miRNAs as candidates for the regulation of immune responses against Gram-negative bacterial infection in *Gallus gallus*.

## 2. Materials and Methods

### 2.1. Cell Culture and LPS Stimulation

HD11 cells, from the cell bank of the Chinese Academy of Sciences, were grown at 37 °C in a humidified atmosphere with 5% CO_2_, supplemented with 10.0 mM HEPES, 1% minimal essential medium non-essential amino acids, 1% glutamine, 10% fetal bovine serum, and 5% chicken serum. When HD11 cells were grown to 80–90% density in 10-cm cell culture dishes, we added 100 ng/mL LPS (Sigma-Aldrich, Saint Louis, MO, USA) from *E. coli* to stimulate the cells in vitro for 6 h before extracting total RNA. LPS-stimulated HD11 cells (L) and unstimulated HD11 cells (C) were harvested. Each group had five biological replicates.

### 2.2. RNA Extraction

To assess the response of transcripts to LPS stimulation in *G. gallus*, we extracted total RNA using TRIzol reagent (Invitrogen Life Technologies, Carlsbad, CA, USA). The total RNA quantity and purity were analyzed using the NanoDrop ND-2000 spectrophotometer (Thermo Scientific, Waltham, MA, USA), and the integrity was checked by Agilent 2100 bioanalyzer (Thermo Scientific) with a minimum RNA integrity number (RIN) of 8.9.

### 2.3. The mRNA Library and Small RNA Preparation and Sequencing

Ten sequencing libraries were constructed by removing rRNA and sequencing on the Illumina NovaSeq 6000 in Novogene (Beijing, China). Five libraries were from the non-LPS group, and another five were from the LPS group. After removing adaptor sequences, low-quality reads, and poly-N reads, the clean reads were mapped to *G. gallus* by Tophat2 and assembled by StringTie. 

The miRNA library was prepared by NEBNext Multiplex small RNA Library Prep Set for Illumina (Set 1) to add the adaptors to the two ends of small RNA. We utilized the particular structure of small RNA (5′ end with complete phosphate groups, 3′ end with hydroxyl radicals) and added the adaptors to both termini and reverse transcribed to cDNA. After polymerase chain reaction (PCR) amplification and PAGE, the cDNA library was obtained. The miRNA library was sequenced on Illumina NovaSeq 6000 (Illumina, San Diego, CA, USA). The raw data were further processed to remove the low-quality reads. We screened the miRNA according to the length of the transcripts, selecting the interval between 18 and 26 nt. We blasted the reads to *G. gallus* by bowtie based on the miRbase to identify the known miRNAs. The ncRNAs were compared with Rfam v.10.1, and we filtered out the rRNA, tRNA, snRNA, and snoRNA. We also filtered out the repeats and reads that were aligned to the exons and introns of mRNA. We predicted the new miRNA by miREvo and mirdeep2 [21,22].

### 2.4. Differential Expression Analysis and Functional Enrichment Analysis

We used StringTie to quantify the mRNA-level read count information and fragments per kb of transcript per million fragments mapped. Subsequently, edgeR was applied to perform the differential expression analysis based on the RNA-seq data. Differentially expressed genes (DEGs) were identified by |log_2_foldchange| ≥ 1 and the corrected *p* (padj) < 0.05. We performed gene ontology (GO) enrichment analysis and Kyoto Encyclopedia of Genes and Genomes (KEGG) pathway analysis for DEGs. The GO terms with *p* ≤ 0.05 were regarded as significantly enriched. Three ontologies showed us molecular function, cellular component, and biological process. For the KEGG pathways, *p* < 0.05 was considered to be significant.

For miRNAs, the expression level was normalized by transcript per million (TPM), the number of reads compared to each miRNA/total ratio of samples to the number of reads × 10^6^. We predicted the biological function of differentially expressed (DE)-miRNAs by the target gene. Only target DEGs that were predicted by both RNAhybrid and miRanda for all of the DE-miRNAs were considered further. GO and KEGG analyses were carried out to determine the biological functions. For an intuitive representation of the relation network between gene and miRNA, we constructed the regulation pattern with cytoscape2.

### 2.5. Real-Time Quantitative PCR of mRNA and miRNAs

We choose the four mRNAs and four miRNAs to confirm the sequencing results. Real-time quantitative PCR was performed on a QuantStudio™ real-time PCR system (Applied Biosystems, Foster City, CA, USA). The primers were designed by Oligo 6.0 and synthesized by the BGI (Beijing Genomics institution, Beijing, China). All of the primers used in the validation assays are listed in Supplementary Materials Table S1. We used PowerUp™ SYBR™ Green Master Mix (Applied Biosystems) to make a 10 μL PCR system, which consisted of 5 μL PowerUp™ SYBR™ Green Master Mix (2×), 0.3 μL upstream and downstream primers for each, 3 μL cDNA template, and 4.4 μL deionized distilled water. The reaction conditions were as follows: 2 min at 50.0 °C, 2 min at 95.0 °C, 40 cycles for 15 s at 95 °C, 15 s at 60 °C, and 1 min at 72 °C. 

## 3. Results

### 3.1. Transcriptome Library Construction

In total, 102.51 million clean reads were obtained, of which 97.49% could be aligned to the reference chicken genome (*G. gallus* 6.0). A large fraction (38.62%) of all mapped sequence reads were located in exon regions. After assembling all the reads from 10 samples, we obtained 51,475 transcripts and 26,163 gene loci. 

In 10 miRNA datasets, we identified 12.67 million high-quality clean reads, and 325,546 unique reads in the range of 18–26 nt in the 10 libraries were obtained from cell samples. After mapping to chicken precursors in miRbase, 680 known miRNAs and 147 novel miRNAs were detected in HD11 cells.

### 3.2. DE-mRNAs and Functional Annotation

**Figure 1:**
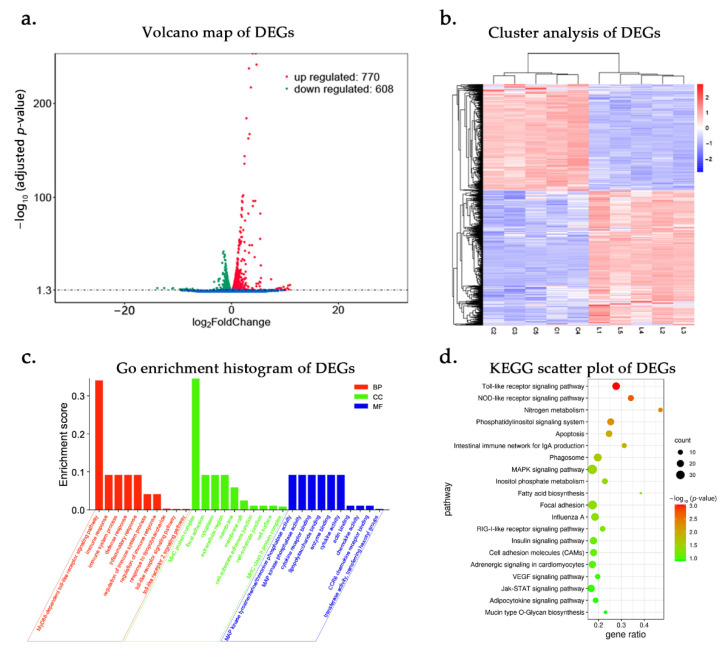
DE-mRNAs and functional annotation after LPS stimulation for 6 h. L represents LPS-stimulated HD11 cells and C represents unstimulated HD11 cells. (**a**) The volcano map of DEGs. (**b**) Heat map plots for DEGs between the control group and LPS stimulation group at 6 h post infection. Sample names are represented in columns and significant genes in rows. Genes are clustered together based on their expression similarity. The color indicates the Z score from high (red) to low (blue). (**c**). GO analysis of the DEGs after LPS stimulation for 6 h. The enrichment score = the number of DEGs in the term ^*^2/number of DEGs in all terms. (**d**) KEGG pathway analysis of DEGs. The vertical axis represents different pathways and the abscissa represents the proportion of significantly differentially expressed genes in the corresponding pathway in all genes in the pathway. The size of the circle represents the number of genes enriched in the corresponding pathway. The larger the circle, the more genes are enriched in the pathway.

We detected 15,883 genes, and 1378 DEGs (DESeq2 with adjusted adjusted *p*-value < 0.05; Supplementary Materials Table S2) were identified in response to LPS during a 6 h time course. Compared to the C group, there were 770 genes up-regulated and 608 down-regulated. The volcano map can visually show the overall distribution of genes with significant differences in expression. Genes with similar expression patterns were clustered together through cluster analysis (Figure 1a). Samples with the same treatment had similar expression profiles and were clustered, reflecting the reliability of the experiment (Figure 1b).

GO analysis of DEGs showed the three categories: biological processes, cellular components, and molecular functions. DEGs were enriched to 8558 GO terms and significantly enriched to 355 terms (adjusted *p*-value < 0.05; Supplementary Materials Table S3). A total of 235 genes were predicted to be related to 59 immunological processes and 6 LPS-related responses, such as cell-mediated immunity (T cells, leukocytes, lymphocytes, etc.), inflammatory response, interleukin production, innate immune response, and adaptive immune response, including some important immune signaling pathways, such as the TLR signaling pathway, TLR2 signaling pathway, and MyD88-dependent TLR signaling pathway (Figure 1c). The TLR signaling pathway plays a pivotal role in defense against LPS challenge and induces downstream inflammatory factor production. The MyD88-dependent TLR signaling pathway is one of the most important pathways for the regulation of the inflammatory response. The MyD88-dependent TLR signaling pathway is critical for regulating NF-κB signaling and innate immune response in *Salmonella* infection. As a central adaptor, MyD88 mediates the initiation of the innate immune response and the production of the proinflammatory cytokines that restrain pathogens and activate adaptive immunity [23]. KEGG analysis demonstrated that DEGs were enriched in 139 pathways, and 137 DEGs were significantly enriched in 12 pathways (*p* < 0.05; Supplementary Materials Table S4). The pathways related to immune response were the TLR signaling pathway, nucleotide oligomerization domain (NOD)-like receptor signaling pathway, and mitogen-activated protein kinase (MAPK) signaling pathway, and they played important roles in LPS infection [24,25,26] (Figure 1d). These signaling pathways showed that, after LPS infection, several DEGs were mainly related to immune function, such as up-regulated expression of CCL5, IL8, NFKBIA, TRAF3, and TNFAIP3, and down-regulated expression of MEF2C, MAP2K6, TLR1, TLR2, and IRF5. They participated in regulating immune-related signaling pathways in chicken macrophage response to LPS.

### 3.3. DE-miRNAs and Functional Annotation

**Figure 2:**
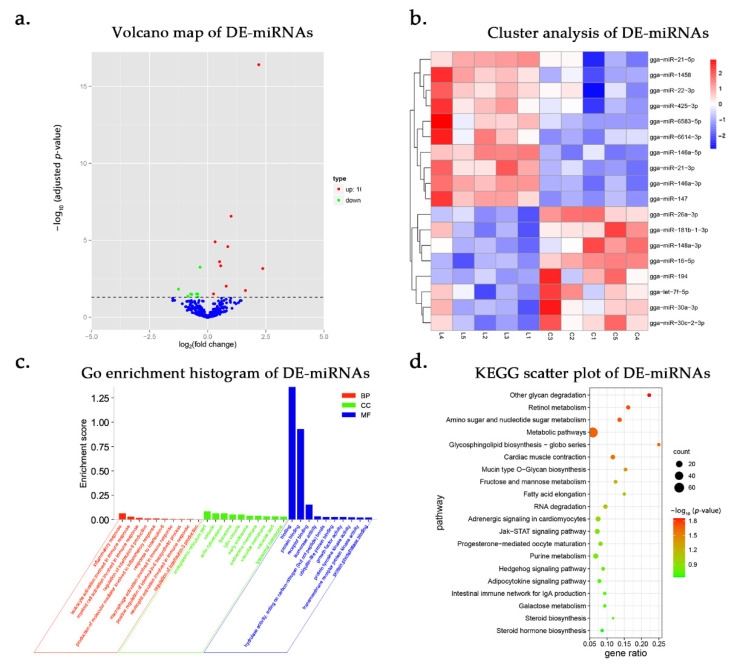
DE-miRNAs and functional annotation after LPS stimulation for 6 h. L represents LPS-stimulated HD11 cells and C represents unstimulated HD11 cells. (**a**) The volcano map of DE-miRNAs. (**b**) Heat map plots for DE-miRNAs between the control group (C) and LPS stimulation group (L) at 6 h post infection. Sample names are represented in columns and significant miRNAs in rows. Genes are clustered together based on their expression similarity. The color indicates the Z score from high (red) to low (blue). (**c**) GO analysis of the target genes of DE-miRNAs after LPS stimulation for 6 h. Enrichment score = number of target genes in the term ^*^2/number of target genes in all terms. (**d**) KEGG pathway analysis of target genes of DE-miRNAs. The vertical axis represents different pathways, and the abscissa represents the proportion of significantly differentially expressed genes in the corresponding pathway in all genes in the pathway. The size of the circle represents the number of genes enriched in the corresponding pathway. The larger the circle, the more genes are enriched in the pathway.

In total, 827 miRNAs were acquired and 18 miRNAs were differentially expressed in response to LPS, including 10 up-regulated and 8 down-regulated miRNAs (adjusted *p*-value < 0.05; Figure 2a, b and Supplementary Materials Table S5). Some miRNAs are involved in inflammatory responses. The let-7f-5p regulates the expression of IL-10 by binding to the 3′ UTR of IL-10, and contributes to poly(methyl methacrylate)-induced osteolysis by promoting the M1 polarization of macrophages [27]. The miR-146a-3p drives trophoblast IL-8 secretion through the activation of TLR8 [28]. The miR-30c-2-3p acts on RAB31 and regulates the GLI1 signaling pathway in gastric cancer tumorigenesis and development [29].

Based on both RNAhybrid and miRanda systems, 55 DEGs can be targeted by 14 DE-miRNAs (Supplementary Materials Table S6). We performed GO analysis of the miRNA target genes, and 418 terms were significantly enriched and 23 terms were related to immunological processes (*p* < 0.05; Supplementary Materials Table S7). 29 target genes were predicted to participate in immunization activities. Most terms are associated with inflammatory factor production, such as the regulation of IL-8 production and the positive regulation of the IL-6 biosynthetic process and the IL-3 biosynthetic process. Immune-related processes such as bone marrow cell activation, neutrophil activation, and macrophage activation were significantly enriched (Figure 2c). KEGG pathway analysis showed that the target genes were mainly enriched in metabolism-related pathways (*p* < 0.05; Figure 2d and Supplementary Materials Table S8).

### 3.4. Correlation Analysis of mRNA and miRNA

**Figure 3:**
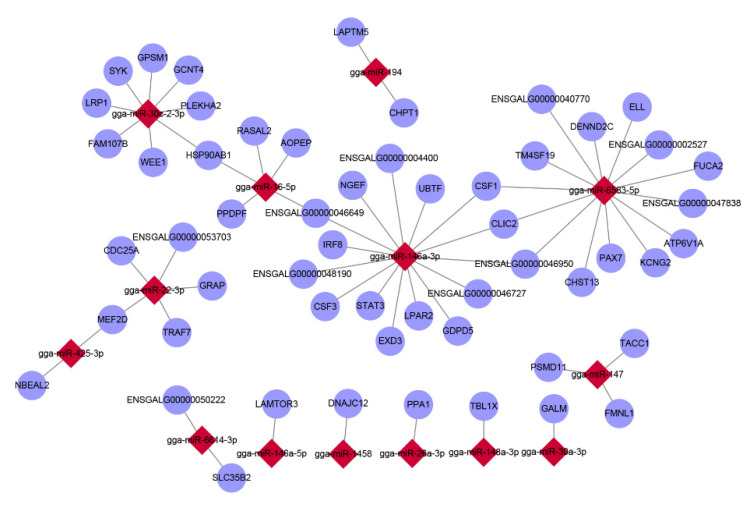
Interaction pattern of DEGs and DE-miRNAs. Taking the intersections of DEGs and target genes of DE-miRNAs, we constructed the interaction network between DEGs and DE-miRNAs by cytoscape2.

We further investigated gga-miR-146a-3p, gga-miR-6583-5p, and gga-miR-30c-2-3p because they were predicted to target many genes that were enriched in immune-related GO terms. The gga-miR-146a-3p was the most differentially expressed miRNA, and may have an essential defense against LPS. The gga-miR-146a-3p targeted a few immune-related genes, such as *IRF8*, *CSF1*, *CSF3,* and *STAT3* (Figure 3). IRF8 is required for the optimal activation of the NLRC4 (NLR family CARD domain containing 4) and NLRP3 (NLR family pyrin domain containing 3) inflammasome activation after infection with Gram-negative bacteria to initiate inflammatory responses [30,31]. TLR4 deficiency results in the down-regulated expression of *CSF3*, *IL6*, and *CCL2* to aggravate dextran–sulfate–sodium-induced intestinal injury [32].

### 3.5. Quantitative Real-Time PCR of mRNAs and miRNAs

**Figure 4:**
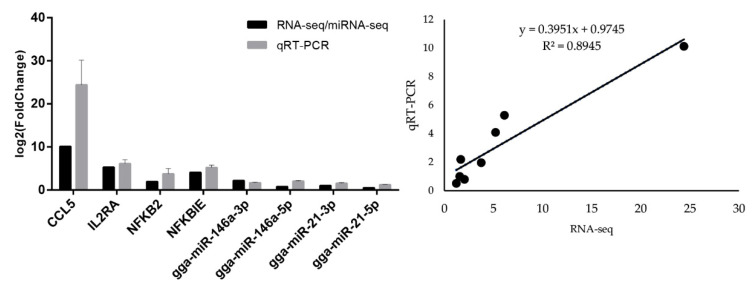
Quantitative real-time PCR of mRNAs and miRNAs compared with the sequencing results. The differences in expression are shown as log_2_(fold change).

Four immune-related DEGs and four DE-miRNAs were selected for quantitative real-time PCR detection: CCL5, IL2RA, NFKB2, NFKBIE, gga-miR-146a-3p, gga-miR-146a-5p, gga-miR-21-3p, and gga-miR-21-5p. We validated the reliability of RNA-seq and miRNA-seq results in the expression regulation of the antibacterial immune response. The significance passed the *t*-test with adjusted *p*-value < 0.05. As shown in Figure 4, the PCR results were in agreement with the RNA-seq/ miRNA-seq data, and the genes/miRNAs were identified as differentially expressed, confirming that the sequencing results were reliable and effective. Pearson’s correlation of the fold-changes between PCR and RNA-seq was 0.95. Overall, the RNA-seq results were considered to be reliable and appropriate for further analysis.

## 4. Discussion

LPS from Gram-negative bacteria is one of the most potent triggers of innate immunity and is often used to simulate Gram-negative bacterial infection. In this study, we used LPS from *E. coli* to explore the role of mRNAs and miRNAs in the host response to LPS infection in *G. gallus* and as a reference for *S. Enteritidis* infections. We applied the RNA-seq and miRNA-seq analyses to understand the pathogenesis of LPS more comprehensively. To the best of our knowledge, there have been few studies on LPS infection in chickens by association analysis of mRNA and miRNA. Therefore, this study can provide a basis for the identification of essential candidate mRNAs and miRNAs.

**Figure 5:**
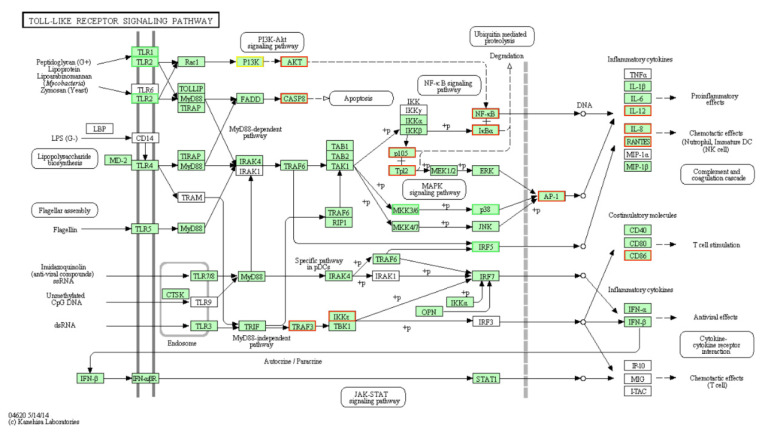
TLR signaling pathway. Red, significantly up-regulated DEGs. Green, significantly down-regulated DEGs. Light green represents the genes involved in the pathway.

We found that most genes were significantly enriched in immunological processes, such as the TLR signaling pathway, TLR2 signaling pathway, and MyD88-dependent TLR signaling pathway. It suggested that TLRs play a central role in chicken pathogen recognition and host–pathogen interactions. Different from the human TLR family, only 10 TLRs have been found in chickens, including two TLR2 isoforms (chTLR2 types 1 and 2); two TLR1/6/10 orthologs; and a single chTLR3, chTLR4, chTLR5, and chTLR7 [33]. In addition, chickens have two TLRs that appear absent in mammalian species, chTLR15 and chTLR21 [34]. In the past, studies on the chicken TLR signaling pathway have focused on TLR4. In this study, the TLR2 signaling pathway (GO:0034134) was mainly enriched, which confirmed the essential role of TLR2. Stimulation of HD11 cells with PmpD-N provoked the secretion of Th2 cytokines IL-6 and IL-10 and up-regulated the expression of TLR2, TLR4, MyD88, and NF-κB, and was regulated by the TLR2/MyD88/NF-κB pathway [35]. We also emphasized the importance of the TLR2 receptor in chickens. Additionally, in the Toll-like receptor signaling pathway, the DEGs mainly participated in the activities of the downstream pathway of TLR1, TLR2, TLR3, and TLR4 (Figure 5). These results suggested that multiple TLR signaling pathways had cross-talk and cascade control for the resistance to LPS.

**Figure 6:**
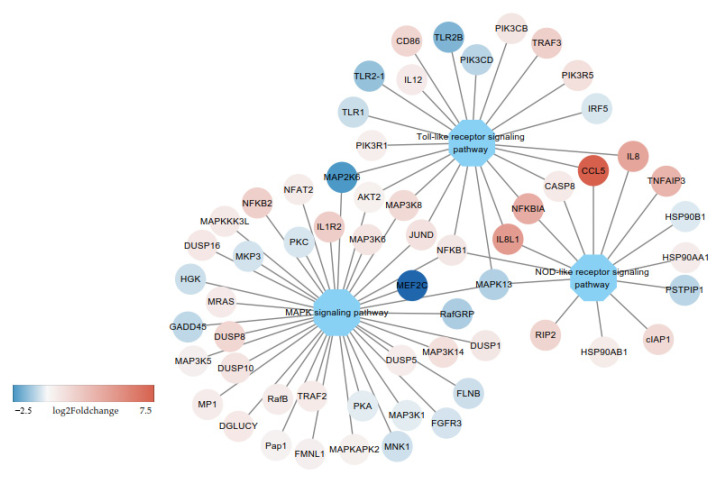
Interaction among several signaling pathways involved in susceptibility to LPS infection. Colors from red to blue represent the fold change (log_2_Foldchange 7.5 to −2.5) of genes between the control group (C) and LPS stimulation group (L). Red represents upregulation and blue represents downregulation. Ellipses represent the genes and octagons represent the pathways.

After chicken macrophages were challenged with LPS, the significantly changed pathways included the TLR signaling pathway, NOD-like receptor signaling pathway, and MAPK signaling pathway. Many immune-related pathways have been identified in previous studies. Our results suggested that multiple signaling pathway cascades controlled the inflammatory response and clearance of foreign pathogens. In the process, CCL5 is the most significantly differentially expressed up-regulated gene and participates in the TLR signaling pathway and NOD-like receptor signaling pathway (Figure 6). CCL5 is a member of the CC subfamily (involved in immunoregulatory and inflammatory processes), which regulates the activation of T-cell expression and secretion. CCL5 is thought to act by promoting leukocyte infiltration to sites of inflammation [36]. Myocyte enhancer factor 2C (MEF2C)is a transcription factor that regulates angiogenesis in endothelial cells. MEF2C restrains the proinflammatory molecules induced by TNF-α, the activation of NF-κB, and leukocyte adhesion to endothelial cells [37].

After screening the miRNA results, we obtained 18 DE-miRNAs, such as gga-miR-146a-3p, gga-miR-21-3p, and gga-miR-22-3p. Many miRNAs have been reported in inflammatory responses. The miR-146a plays a role in regulating the expression of IL-1 receptor-associated kinase and TNF-receptor-associated factor 6 after LPS infection [38]. In a human umbilical vein endothelial cell angiogenesis study, the knockdown of miR-146a activated transforming growth factor-β1 signaling to inhibit angiogenesis [39]. Recently, the miR-21-3p was verified to target RGS4 and attenuate inflammatory responses and apoptosis caused by LPS in ARPE-19 cells [40]. 

When we constructed an interaction network between DEGs and DE-miRNAs, three miRNAs that were predicted to target several genes were highlighted to have a vital role in response to LPS, namely gga-miR-146a-3p, gga-miR-30c-2-3p, and gga-miR-6583-5p. It is worth mentioning that gga-miR-146a-3p was the most significant DE-miRNA after LPS stimulation. Interferon regulatory factors (IRFs) are a family of transcription factors that regulate many aspects of innate and adaptive immune responses, including antiviral responses, responses to pathogens, proinflammatory responses, and regulating immune cell differentiation [41]. IRF8 is required for the development of lymphoid and myeloid cells (dendritic cells, monocytes, and macrophages). IRF8 plays a critical role in immune cell functions, protection against infections, and susceptibility to inflammatory diseases [42]. IRF8 is also involved in the MyD88-mediated NF-κB signaling pathway. In teleost fish, IRF8 and IRF3 regulate MyD88 in different ways; IRF3 promotes the MyD88-mediated NF-κB signaling pathway, whereas IRF8 inhibits the signaling pathway [43]. Thus, IRF8-deficient macrophages are susceptible to ex vivo infection with *Mycobacterium bovis*, *Salmonella typhimurium*, and *Legionella pneumophila*. Some studies have shown that IRF8 synergizes with its partner, IRF1, in the IFN-γ-dependent activation of intrinsic macrophage antimicrobial defenses, the production of key inflammatory cytokines activating early immune responses (IL-12p40, IL-18, RANTES, and TNF-α), and the expression of genes involved in the maturation of myeloid (DCs) and lymphoid (NK, CD8+ T cells) cells [44]. Hence, the IRF8– miR146a-3p target pair deserves further attention.

## 5. Conclusions

We performed a transcriptional analysis, and mainly focused on DE-mRNAs and DE- miRNAs of chicken macrophages in response to LPS. In total, 1378 DE-mRNAs and 18 miRNAs were identified. 235 genes may be related to immunological processes and LPS-related responses. The main signaling pathway was the TLR signaling pathway, which suggested a crucial role of TLR2 in response to pathogens in chickens. Based on the prediction of the target gene, 55 DEGs can be targeted by 14 DE-miRNAs and 29 target genes were predicted to participate in immunization activities. In the interaction network between DEGs and DE-miRNAs, gga-miR-146a-3p, gga-miR-30c-2-3p, and gga-miR-6583-5p were predicted to target 34 genes that were highlighted to have a vital role in the response to LPS. Our results provide a foundation for the study of LPS infection in chicken macrophages, an essential reference for a better understanding of *S. Enteritidis*, and further information about the mechanisms of host resistance and susceptibility to LPS infection in chickens.

## Data Availability

The RNA-Seq and miRNA-Seq raw data in this paper have been deposited in the Genome Sequence Archive in BIG Data Center Members, Beijing Institute of Genomics (BIG), Chinese Academy of Sciences, under the accession numbers of CRA004161 and CRA004160 that are publicly accessible at https://bigd.big.ac.cn/gsub/ (accessed on 27 April 2021).

## Supplementary Materials

The following are available online at https://www.mdpi.com/article/10.3390/genes12050760/s1: Table S1: The primers used in the validation assays. Table S2: The expression of genes. Table S3: GO analysis of the DEGs. Table S4: KEGG pathway analysis of the DEGs. Table S5: The expression of miRNA. Table S6: The target gene of miRNA. Table S7: GO analysis of the target genes of DE-miRNAs. Table S8: KEGG pathway analysis of the target genes of DE-miRNAs.
